# Targeting the formation of estrogens for treatment of hormone dependent diseases–current status

**DOI:** 10.3389/fphar.2023.1155558

**Published:** 2023-04-28

**Authors:** Tea Lanišnik Rižner, Andrea Romano

**Affiliations:** ^1^ Laboratory for Molecular Basis of Hormone-Dependent Diseases and Biomarkers, Institute of Biochemistry and Molecular Genetics, Faculty of Medicine, University of Ljubljana, Ljubljana, Slovenia; ^2^ GROW Department of Gynaecology, Faculty of Health, Medicine and Life Sciences (FHML)/GROW-School for Oncology and Reproduction, Maastricht University, Maastricht, Netherlands

**Keywords:** intracrinology, estrogens, breast cancer, ovarian cancer, endometrial cancer, endometriosis, prostate cancer

## Abstract

Local formation and action of estrogens have crucial roles in hormone dependent cancers and benign diseases like endometriosis. Drugs that are currently used for the treatment of these diseases act at the receptor and at the pre-receptor levels, targeting the local formation of estrogens. Since 1980s the local formation of estrogens has been targeted by inhibitors of aromatase that catalyses their formation from androgens. Steroidal and non-steroidal inhibitors have successfully been used to treat postmenopausal breast cancer and have also been evaluated in clinical studies in patients with endometrial, ovarian cancers and endometriosis. Over the past decade also inhibitors of sulfatase that catalyses the hydrolysis of inactive estrogen-sulfates entered clinical trials for treatment of breast, endometrial cancers and endometriosis, with clinical effects observed primarily in breast cancer. More recently, inhibitors of 17beta-hydroxysteroid dehydrogenase 1, an enzyme responsible for formation of the most potent estrogen, estradiol, have shown promising results in preclinical studies and have already entered clinical evaluation for endometriosis. This review aims to provide an overview of the current status of the use of hormonal drugs for the major hormone-dependent diseases. Further, it aims to explain the mechanisms behind the -sometimes- observed weak effects and low therapeutic efficacy of these drugs and the possibilities and the advantages of combined treatments targeting several enzymes in the local estrogen formation, or drugs acting with different therapeutic mechanisms.

## 1 Introduction

### 1.1 Estrogen dependent diseases

**FIGURE 1:**
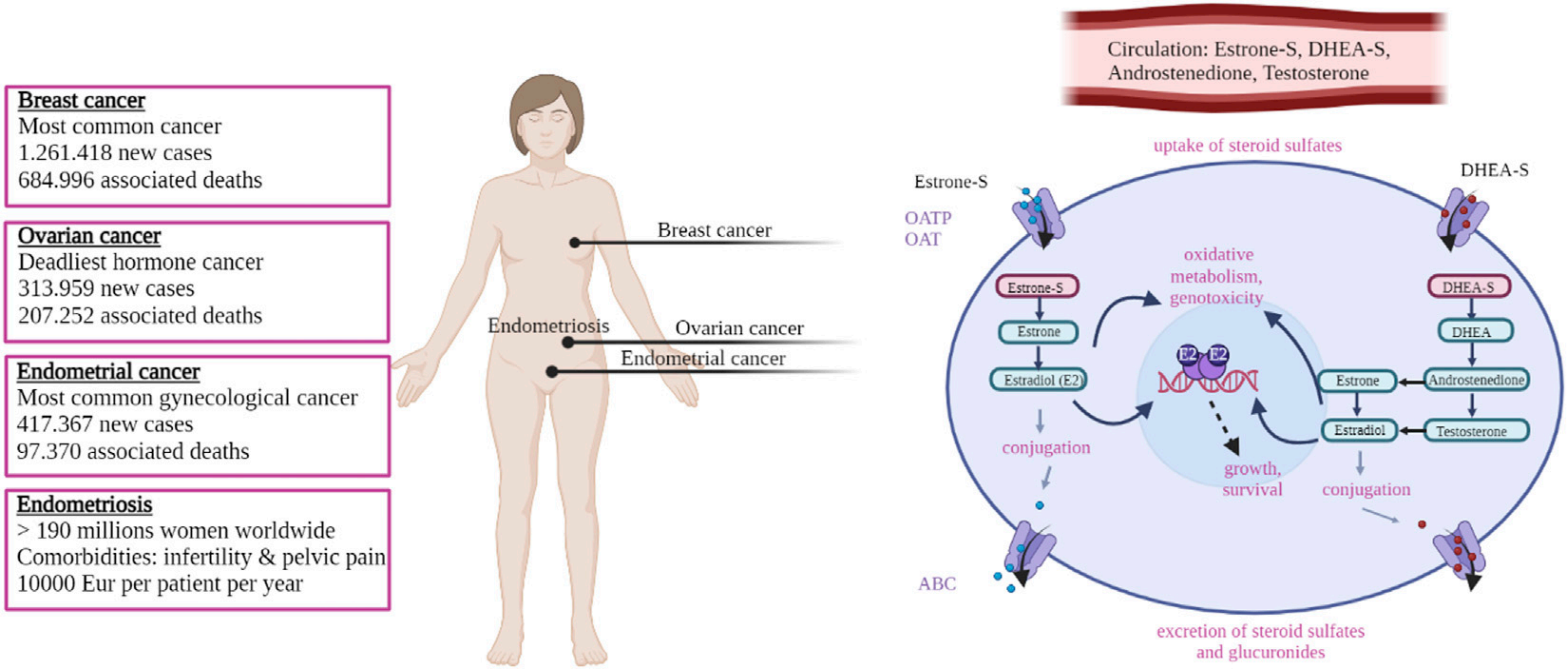
Schematic overview of the most common female hormone dependent diseases and the molecular mechanism leading to estrogen exposure. Boxes on the left report the main figures related to the indicated diseases (see main text for details and references). On the right estrogen signaling is shown schematically; sulfated compounds like estrone-S and DHEA-S are activated by desulfation. Estradiol is the final active estrogenic compound, derived directly from estrone-S but also through other routes via Androstenedione and Testosterone (see text for details). Estradiol binds to and activates the estrogen receptor (ER) and modulate the transcription of target genes, leading to proliferation. Oxidative metabolism of estrogens can also lead to the formation of genotoxic compounds. See text for details. Created in BioRender.com.

Estrogens have important roles in the development of hormone-dependent cancers like breast, endometrial, ovarian cancers (Figure 1) but also prostate cancer. Hormone dependent cancers comprise more than 20% of all cancers worldwide and more than 35% of cancers in women (https://gco.iarc.fr/today, accessed 30 January 2023; (Sung et al., 2021)). Breast cancer is the most common malignancy, with 2.261.419 new cases and 684.996 associated deaths estimated for 2020 worldwide (Sung et al., 2021). Endometrial cancer is the sixth most frequent cancer worldwide, with 417.367 new cases and 97.370 deaths and ovarian cancer is the deadliest hormone dependent cancer with 313.959 new cases and 207.252 deaths, both estimated for 2020 (Sung et al., 2021). Prostate cancer is the second most frequent cancer in men with 1.414.259 new cases and 375.304 deaths estimated for 2020 (Sung et al., 2021). The number of newly identified hormone-dependent cancers cases is increasing every year. For endometrial cancer, European estimates suggest that by 2025 there will be a 50%–100% increase in incidence compared to 2005 (Lindemann et al., 2010). A rising burden of both premenopausal and postmenopausal breast cancer is expected worldwide (Heer et al., 2020) as is the burden of ovarian cancer, the incidence of which is projected to increase by 55% and the number of deaths by 67% by 2035 (World Ovarian Cancer Coalition; https://worldovariancancercoalition.org, accessed 30 January 2023). In case of prostate cancer, although some US projections expect a decrease in number of cases by 2040 (Rahib et al., 2021), other country-specific estimates project a higher number of cases in future, with Germany predicting that prostate cancer will be the most common malignancy by 2030, thus surpassing breast cancer (Quante et al., 2016).

Hormone-dependent diseases include also a number of non-cancerous conditions (Figure 1) like endometriosis, uterine fibroids and polycystic ovarian syndrome (PCOS). Although being non-life-threatening, these diseases can be highly prevalent among the population, and can have a strong impact on the private life of the patients and the society. Endometriosis is a highly prevalent condition that affects up to 10% of women in their reproductive age, which sums up to 190 million women worldwide (Chapron et al., 2019; Zondervan et al., 2020). Endometriosis is associated with infertility and chronic pelvic pain. The disease has a strong impact on the patient private and professional life (De Graaff et al., 2013; De Graaff et al., 2016) and a multi-centre study dating over 10 years ago (the EndoCost study) estimates that the yearly disease-costs, associated with healthcare and productivity loss, approximate 10,000€ per patient (Simoens et al., 2012). Uterine fibroids are the most common (benign) neoplasm affecting women in their reproductive age, and they cause significant morbidity, heavy menstrual and abnormal uterine bleeding (Stewart et al., 2017). PCOS is the most common endocrine–metabolic disorder in reproductive-aged women with a prevalence ranging between 5% and 20%, depending on the diagnostic criteria (Azziz, 2018). PCOS is characterised by hyperandrogenism, low fertility and chronic anovulation that also result in hypoprogestogenism and hyperestrogenism (Luan et al., 2022). PCOS has important immediate short-term consequences (like dermatologic, reproductive) as well as several longer-term metabolic, cardiovascular co-morbidities and disorder that include impaired glucose tolerance, type 2 diabetes, metabolic syndrome, non-alcoholic fatty liver disease, dyslipidemia, vascular dysfunction, hypertension (Zore et al., 2017). In addition, the treatment of these diseases in young girls and adolescents is a particular challenge (Chapron et al., 2019; Witchel et al., 2019; Zondervan et al., 2020).

In women, large part of hormone dependent cancers, like endometrial, ovarian and a proportion of breast cancers, occur after menopause when ovaries have ceased to produce steroids. Therefore, these tumours depend on the local (intracrine) estrogen formation from inactive precursors of ovarian or adrenal origin, like dehydroepiandrosterone sulfate (DHEA-S), DHEA, androstenedione and estrone sulfate (estrone-S; (Rizner et al., 2017; Konings et al., 2018b; Cornel et al., 2018; Cornel et al., 2019). These same mechanisms are also implicated in non-oncologic conditions reported above and typical of reproductive age (Rizner, 2009; Konings et al., 2018a).

The term intracrinology was coined by (Labrie, 1991), following his report that in humans and other primates, active steroid hormones can be formed in peripheral tissues from adrenal precursors. He introduced the term intracrinology and intracrine action, which describe the formation of steroid hormones and their action within the same cell. Three key enzymes are responsible for the local (intracrine) formation of the most active estrogen, i.e., estradiol. Aromatase (CY19A1) catalyses the irreversible aromatization of androstenedione and testosterone to estrone and estradiol, respectively. Sulfatase (STS) catalyses the hydrolysis of estrone-S to estrone as well as DHEA-S to DHEA (Rizner, 2016). In contrast to irreversible aromatase pathway, the action of sulfatase is opposed by sulfotransferases (SULTs) that catalyse the sulfation of estrone (SULT1E1) and DHEA (SULT2B1) (Rizner, 2016; Mueller et al., 2021).

Last, the enzyme 17β-hydroxysteroid dehydrogenase 1 (HSD17B1) acts as a 17-hydroxysteroid reductase and has a high catalytic efficiency for the conversion of estrone to the most potent estrogen, estradiol. The action of HSD17B1 is opposed by the oxidative enzyme HSD17B2 responsible for inactivation of estradiol back to estrone (Rizner, 2009; Konings et al., 2018c).

**FIGURE 2:**
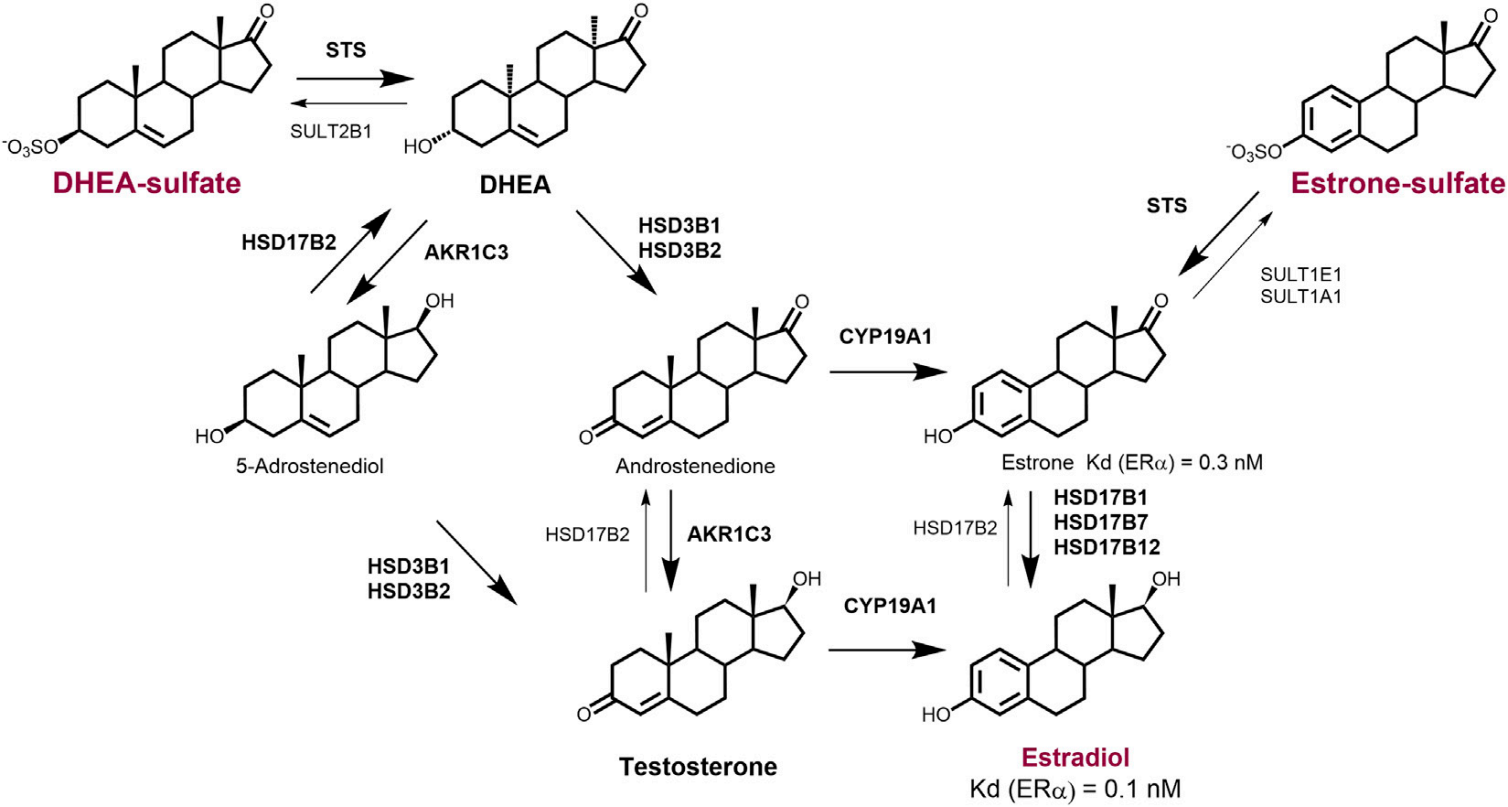
Overview of the major intracrine routes responsible for the generation of local estradiol. Deydroepiandrosterone-sulfate (DHEA-S) and estrone-sulfate (E1-S) serve as precursors for the formation of active steroid hormones. Estradiol (E2) can be formed from circulating DHEA-S or androstenedione by the action of sulfatase (STS), 3β-hydroxysteroid dehydrogenases type 1 and 2 (HSD3B1 and HSD3B2), aromatase (CYP19A1) and reductive 17β-hydroxysteroid dehydrogenase (HSD17B1). Estradiol can also be formed from circulating estrone-S or some levels of estradiol-S by the action of STS and HSD17B1. DHEA-S and androstenedione are formed in the adrenal gland and in ovaries and testes. Estrone-S is formed in adrenal gland and adipose tissue. 5-Androstanediol can serve as an intermediate in the synthesis of testosterone and estradiol in testes and peripheral tissues.

Local/intracrine estrogen biosynthesis thus comprises two complementary pathways, the aromatase pathway from DHEA or androstenedione and the sulfatase pathway from estrone-S (Figure 2). HSD17B1 has crucial role in both pathways. Finally, although steroids can freely diffuse through the cell membrane, sulfated compounds in particular, like estrone-S, enter the cell via facilitated diffusion catalysed by organic anion transporting polypeptides (OATPs) and organic anion transporters (OATs; (Hagenbuch and Meier, 2003; Rizner et al., 2017).

These pathways are not only involved in the intracrine estrogen synthesis in women’s cancer, but they have roles also in prostate cancer, where estradiol can be formed from testosterone produced in Leydig cells or from circulating estrone-S and can, similarly to the situation in female cancers, stimulate proliferation of cancerous cells (Di Zazzo et al., 2018; Ramirez-de-Arellano et al., 2021). Besides cell proliferation, additional effects of estrogens result from their local metabolism to catechols and genotoxic quinones that can form depurinating adducts (Rizner, 2013; Cavalieri and Rogan, 2016).

Due to the central role of estrogens in cell proliferation and due to their potential genotoxicity, blocking the estrogen signalling is a therapeutic strategy for estrogen dependent cancers and non-oncologic conditions. Estrogen action can be blocked at various level: a) at the uptake/efflux level, for instance by interfering with the activity of OAT/OATPs (no compound has currently reached the experimental *in-vivo* phase); however, such strategy in unlikely to be applicable in clinic since studies showed blocking one single OATP is not sufficient to inhibit steroid hormone uptake, but blocking simultaneously multiple OATPs will disturb the metabolic homeostasis and the protection against toxins (Rizner et al., 2017); b) estrogen action can also be blocked at the receptor level, i.e., the drug binds to the estrogen receptor (ER) and blocks/modify its action (like, for instance, fulvestrant, tamoxifen, raloxifen, toremifen); c) or it can be blocked at the pre-receptor (or intracrine) level, where the local generation of estradiol is targeted.

Drugs that are currently used for treatment of hormone-dependent diseases act at the pre-receptor and receptor levels. Among the molecules that act at the receptor level, the selective ER receptor modulator tamoxifen has a central role in the treatment of breast cancer. Tamoxifen was discovered by serendipity by (Jordan, 2021) and was approved by FDA for the treatment of metastatic breast cancer in 1977 and later for the treatment of ER positive breast cancer. Also the selective ER degrader Fulvestrant has an important role in breast cancer treatment (Nathan and Schmid, 2017) and a number of additional novel therapeutic compounds with improved pharmacokinetics act at the receptor level and are under investigation (Furman et al., 2019). These compounds will not be further discussed in the present review, that will rather focus of the molecules targeting the pre-receptor steroid metabolism.

With regard to drugs interfering with estrogen signalling at the pre-receptor level, since pivotal discoveries by (Brodie et al., 1977) the intracrine formation of estrogens has been targeted by inhibitors of aromatase. Steroidal and non-steroidal inhibitors (Brodie, 2002; Smith and Dowsett, 2003; Brodie et al., 2011) have successfully been used for the treatment of postmenopausal breast cancer and have also been evaluated in clinical studies in patients with endometrial and ovarian cancers, patients with endometriosis and also patients with prostate cancer (see below). In the last decade also one of the most potent sulfatase inhibitors, Irosustat, synthetized by Dr. Potter’s group (Purohit et al., 1995) entered the clinical phase for the treatment of breast, ovarian, endometrial and prostate cancers with various levels of therapeutic effects observed. Several compounds with inhibitory action on the enzyme HSD17B1 have been developed and one steroidal compound, a competitive HSD17B1 inhibitor (OG-6219) recently entered the clinical phase for endometriosis (https://www.elenaendometriosisstudy.com/#!/) and it is in the preclinical phase for endometrial and breast cancer (Husen et al., 2006a; Husen et al., 2006b; Konings et al., 2018b; Jarvensivu et al., 2018; Xanthoulea et al., 2021). The first irreversible inhibitor of HSD17B1 developed by Dr. Poirier’s group and Dr. Frotscher’s group is currently being used in the preclinical phase (Salah et al., 2019; Poirier et al., 2022).

In the next sections, we will elaborate on the importance of aromatase, the sulfatase pathways and the HSD17B1-2 redox balance in hormone dependent diseases. We will then continue with an overview of the clinical studies that target the local formation of estrogens. To this end, we searched the Clinical Trial Database from the U.S. National Library of Medicine (ClinicalTrials.gov; accessed on date 25 January 2023) and integrated with PubMed searches. We specifically focussed on breast, endometrial, ovarian, prostate cancers and endometriosis, whereas we will not further discuss uterine fibroids and PCOS since the role of the local steroids in these conditions is underexplored.

## 2 The aromatase pathway in hormone dependent diseases

The gene *CYP19A1* that encodes for the aromatase is expressed in the ovaries, testis, adipose tissue, breast, adrenal gland, skin and some other tissues, although at lower expression levels (Konings et al., 2018a). The aromatase is also expressed in diseased tissues like breast cancer and, although with contrasting data in literature, endometrial cancer (Rizner, 2013; Konings et al., 2018b; Cornel et al., 2019), endometriosis (Rizner, 2009; Huhtinen et al., 2012). *CYP19A1* is also expressed in prostate cancer where it is associated with longer time to disease progression (Grindstad et al., 2016).

Tissue-specific expression of *CYP19A1* is controlled by the use of alternative (tissue-specific) promoters and transcription factors (Simpson and Davis, 2001; Barros-Oliveira et al., 2021). Increased aromatase expression leads to a higher local formation of estrogens, as reported in breast cancer tissue (Friesenhengst et al., 2018; Barros-Oliveira et al., 2021). Aromatase converts androgens to estrogens and depends on local or circulating androgen levels. In postmenopausal women, the circulating levels of androstenedione and testosterone decrease by half approximately compared with the premenopausal state, but these low nM levels of androstenedione (0.84–2.79 nM) and testosterone (0.21–0.83 nM (Krichbaum et al., 1990) are sufficient for local formation of estrogens. Also in middle aged compared with young man, circulating levels of androgens decrease to 3.11–3.35 nM for androstenedione and 16.23–17.48 nM for testosterone (Giton et al., 2011). In addition, estrogens can upregulate *CYP19A1* expression *via* cross-talk with growth factor-mediated pathways (Kinoshita and Chen, 2003) and mediators of inflammation like TNFα, PGE2, IL1β and IL6 (Singh et al., 1999; Gerard and Brown, 2018), thus further sustaining these intracrine pathways.

### 2.1 Overview of aromatase inhibitors

CYP19A1 is the rate-limiting enzyme in the synthesis of estrogens and aromatase inhibitors block its action. Table 1 overviews the most relevant aromatase inhibitors developed.

**TABLE 1:** Overview of the major aromatase inhibitors developed so far.

Compound name	Drug name(s)	Features Mechanism	Note
Aminoglutethimide	Elipten, Cytadren, Orimeten	1^st^ generation non-steroidal	Indicated for Seizures, Cushing’s syndrome, breast and prostate cancer.
		non-selective*	Withdrawn during the ‘60s due to toxicity
Testolactone	Teslac	steroidal	Indicated for breast cancer.
		irreversible	Discontinued (2008)
		non-selective*	
Vorozole	R-76713	non-steroidal	Stopped during the clinical testing, non-superiority**
	Rivizor	reversible	
		competitive	
Formestane	Lentaron	2^nd^ generation steroidal	Indicated for breast cancer.
			Discontinued (low potency, lack of specificity, side effects)
Fadrozole	Afema	2^nd^ generation non-steroidal	Discontinued (low potency, lack of specificity, side effects)
Atamestane		steroidal	Discontinued for Benign prostatic hyperplasia; Breast cancer
		irreversible	
MDL-18962	Plomestane	steroidal	Indicated for breast cancer.
		irreversible	Clinical development halted for technical reasons
Anastrozole	Arimidex	3^rd^ generation non-steroidal	In use for breast cancer
		reversible	
		competitive	
Letrozole	Femara	3^rd^ generation non-steroidal	In use for breast cancer
		reversible	
		competitive	
Exemestane	Aromasin	3^rd^ generation steroidal	In use for breast cancer
		irreversible	

*Inhibitor of various CYP, enzymes, including aromatase, ** (Goss, 1998).

Type 1 inhibitors are steroidal compounds that bind irreversibly to the enzyme. Most type 1 aromatase inhibitors developed initially consisted of the steroid scaffold of androstenedione with modifications in the A or B steroidal rings. From 2000, several research teams explored aromatase inhibitors with novel chemistry, based on D ring modifications and the exploration for novel compounds is today very active. Non-steroidal aromatase inhibitors, or type 2, interact with the heme-moiety of the CYP enzyme, and act as reversible, competitive inhibitors. Type 2 aromatase inhibitors are chemically derived from imidazole and, similar to the type 1 inhibitors, research is continuously exploring novel compounds. For an overview on the numerous promising compounds and their chemistry we redirect the readers to a comprehensive review (Ahmad and Shagufta, 2015).

An additional class of aromatase inhibitors is represented by natural compounds with phytoestrogenic properties. These molecules are however outside the scope of the present review and will not be further discussed. We would like to redirect eventually interested readers to recent reviews (Bolton et al., 2019; Nielsen and McNulty, 2019).

The aromatase inhibitors that are currently used in clinic are referred to as third generation aromatase inhibitors and are letrozole, anastrozole (both type 2, non-steroidal) and exemestane (type 1, steroidal compound; Table 1).

### 2.2 Clinical trials targeting aromatase

Although first and second generation aromatase inhibitors had little therapeutic efficacy and relatively high toxicity (Hong et al., 2009), a number of studies demonstrated that third generation compounds are efficient and superior to tamoxifen (standard hormonal drug at the time) and these studies represented the milestones that led to the approval of third generation aromatase inhibitors for the treatment breast cancer by FDA/EMA (Baum et al., 2003; Goss, 2003; 2007; Coombes et al., 2004; Goss et al., 2005; Jonat et al., 2006; Coates et al., 2007; Gibson et al., 2007; Mamounas et al., 2008).

In the following overview, only clinical trials that are completed are considered (whereas those that are still running, terminated are excluded).

#### 2.2.1 Aromatase inhibitors and breast cancer

In total, 452 completed clinical trials based on the use of aromatase inhibitors for breast cancer are currently registered in the database Clinical Trials, and for 163 trials results are available, including those that have led to the approval of the third generation aromatase inhibitors in current clinical practice and are briefly discussed above. Since the clinical trials investigating the efficacy of aromatase inhibitors in breast cancer have been the focus of several recent reviews (Riemsma et al., 2010; Wang et al., 2022), and since the use of aromatase inhibitors as adjuvant therapy and in metastatic breast cancer is well established (Chumsri et al., 2011; Maugeri-Sacca et al., 2014; Waks and Winer, 2019; Kharb et al., 2020), recently also in the extended protocol (Goodwin, 2021), we will only consider the trials that explored the therapeutic effect of aromatase inhibitors in combination with other targeted drugs (and therefore we excluded the combination of aromatase inhibitors with other hormonal drugs, chemotherapy, external beam radiotherapy, with drugs alleviating symptoms of aromatase inhibitor and food supplements).

In the last two decades, a number of trials were initiated that evaluated the effects of aromatase inhibitors in combination with other targeted drugs, with 153 trials registered in the database Clinical Trials, and 59 trials with results (Supplementary Table S1). Among these, 37 are phase 2 studies that tested the therapeutic efficacy of the inhibition of aromatase in combination with drugs targeting vascular endothelial growth factor (VEGF), insulin-like growth factor (IGF), mammalian target of rapamycin (mTOR), histone-deacetylases (HDAC), epidermal growth factors receptors (EGFR and HER2), cyclo-oxygenase (COX-2), cyclin-dependent and tyrosine kinases (CDK and TK). Of these molecules targeting CDK, HER2, VEGF and mTOR proceeded through phase 3 (10 studies in total) and phase 4 (two studies) and demonstrated the efficacy and the safety of these drug regimens (Baselga et al., 2012; Krop et al., 2014; Noguchi et al., 2014; Kim et al., 2016; Krop et al., 2017; Johnston et al., 2018; Martin et al., 2019; Lynce et al., 2021); see Supplementary Table S1 for further details.

#### 2.2.2 Aromatase inhibitors and endometrial cancer

There are 28 trials where an aromatase inhibitor has been evaluated in patients with endometrial cancer, nine have been completed and for four trials results are available (Table 2).

**TABLE 2:** Overview of the trials registered in the database Clinical Trials (ClinicalTrails.gov) for the use of aromatase, STS and HSD17B1 inhibitors in breast, endometrial, ovarian, prostate cancers and endometriosis.

Condition	Number References	Title		Acronymn	Status	Res.	Drug(s)	Sponsor
Aromase INHIBITORS (only completed/with results; for breast cancer refer to Supplemental Table I)								
Endometrial Cancer	NCT01068249 (Slomovitz et al., 2015)		Letrozole and RAD001 With Advanced or Recurrent Endometrial Cancer		Active, not recruiting	yes	Letrozole|RAD001 (Everolimus)	M.D. Anderson Cancer Center|Novartis
Endometrial Cancer	NCT02228681		Everolimus and Letrozole or Hormonal Therapy to Treat Endometrial Cancer		Active, not recruiting	yes	Everolimus|Tamoxifen|Letrozole|Medroxyprogesterone Acetate	Gynecologic Oncology Group|Novartis Pharmaceuticals|GOG Foundation
Endometrial Cancer	NCT00997373		Letrozole as a Treatment of Endometrial Cancer		Completed	yes	Letrozole	University of California, Davis
Endometrial Cancer	NCT02657928 (Colon-Otero et al., 2020)		Ribociclib and Letrozole in Treating Patients With Relapsed ER Positive Ovarian, Fallopian Tube, Primary Peritoneal, or Endometrial Cancer		Completed	yes	Letrozole|Ribociclib	Mayo Clinic|National Cancer Institute (NCI)
Ovarian Cancer	NCT02101788 (Gershenson et al., 2022)		Trametinib in Treating Patients With Recurrent or Progressive Low-Grade Ovarian Cancer or Peritoneal Cavity Cancer		Active, not recruiting	yes	Letrozole|Paclitaxel|Pegylated Liposomal Doxorubicin Hydrochloride|Tamoxifen Citrate| Topotecan|Trametinib dimethyl sulfoxide	National Cancer Institute (NCI)|NRG Oncology
Ovarian Cancer	NCT02657928 (Colon-Otero et al., 2020)		Ribociclib and Letrozole in Treating Patients With Relapsed ER Positive Ovarian, Fallopian Tube, Primary Peritoneal, or Endometrial Cancer		Completed	yes	Letrozole|Ribociclib	Mayo Clinic|National Cancer Institute (NCI)
Ovarian Cancer	NCT02283658		Everolimus and Letrozole in Treating Patients With Recurrent Hormone Receptor Positive Ovarian, Fallopian Tube, or Primary Peritoneal Cavity Cancer		Completed	yes	Everolimus|Letrozole	Mayo Clinic|National Cancer Institute (NCI)
Ovarian Cancer	NCT00505661		Letrozole in Patients With Ovarian Tumors		Terminated	yes	Letrozole	M.D. Anderson Cancer Center
Endometriosis	NCT02203331		Bay98-7196, Dose Finding/POC Study		Completed	yes	Placebo|Levonorgestrel|Anastrozole|Lupron/Leuprolide acetate	Bayer
STS INHIBITORS (all studies)								
Breast Cancer	NCT01785992		A Study of the Safety and Effectiveness of Irosustat When Added to an AI in ER + ve Locally Advanced or Metastatic Breast Cancer.	IRIS	Completed	no	Irosustat	Imperial College London|Cancer Research United Kingdom
Breast Cancer	NCT01230970		Exploratory Study to Assess the Short Term Intratumoural and Peripheral Effects of BN83495 in Postmenopausal Women With Newly Diagnosed Breast Cancer		Terminated	no	BN83495	Ipsen
Breast Cancer	NCT01662726		A Study to Assess the Ability of a Novel Endocrine Treatment for Breast Cancer, Irosustat, to Slow Down Cancer Growth	IPET	Terminated	no	Irosustat	Imperial College London|National Institute for Health Research, United Kingdom|Ipsen|Imperial College Healthcare NHS Trust|Guy’s and St Thomas’ NHS Foundation Trust|University of Southern California|QPS Netherlands B.V.
Breast Cancer	NCT01840488		BN83495 Phase I in Post-menopausal Women		Completed	no	Irosustat (BN83495)	Ipsen
Breast Cancer	NCT00397501		BBBD Followed By Methotrexate and Carboplatin With or Without Trastuzumab in Treating Women With Breast Cancer That Has Spread to the Brain		Withdrawn	no	trastuzumab| carboplatin|methotrexate|sodium thiosulfate	OHSU Knight Cancer Institute|National Cancer Institute (NCI)
Breast Cancer	NCT03905343		Ribociclib-endocrine Combination Therapy Versus Chemotherapy as 1st Line in Visceral mBC		Terminated	no	Ribociclib|Mono-chemotherapy|Endocrine-Therapy	Swiss Group for Clinical Cancer Research|The Belgian Society of Medical Oncology
Breast Cancer	NCT04961996		A Study Evaluating the Efficacy and Safety of Adjuvant Giredestrant Compared With Physician’s Choice of Adjuvant Endocrine Monotherapy in Participants With Estrogen Receptor-Positive, HER2-Negative Early Breast Cancer (lidERA Breast Cancer)		Recruiting	no	Giredestrant|Endocrine Therapy of Physician’s Choice|LHRH Agonist	Hoffmann-La Roche
Endometrial Cancer	NCT00910091		The Study of Oral Steroid Sulphatase Inhibitor BN83495 Versus Megestrol Acetate (MA) in Women With Advanced or Recurrent Endometrial Cancer		Completed	yes	BN83495|Megestrol Acetate (MA)	Ipsen
Endometrial Cancer	NCT01251354		Study of BN83495 in Post-menopausal Women With Endometrial Cancer Post-chemotherapy		Terminated	yes	BN83495	Ipsen
Endometriosis	NCT01631981		PGL2001 Proof of Concept Study in Symptomatic Endometriosis	AMBER	Completed	no	PGL 2001 + Primolut-Nor 5|Drug: Placebo + Primolut-Nor 5	PregLem SA
Prostate Cancer	NCT00790374		BN83495 in Prostate Cancer	STX64PC	Completed	no	BN83495 (Cohort 1)|BN83495 (Cohort 2)|BN83495 (Cohort 3)	Ipsen
HSD17B1 INHIBITORS (all studies)								
Endometriosis	NCT05560646		A Study to Investigate Efficacy and Safety of OG-6219 BID in 3 Dose Levels Compared With Placebo in Participants Aged 18 to 49 With Moderate to Severe Endometriosis-related Pain	ELENA	Recruiting	no	Drug: OG-6219|Drug: Placebo	Organon and Co.|Iqvia Pty Ltd.
Endometriosis	NCT03709420		A Study to Investigate the Safety, Tolerability, Food Effect, Pharmacokinetics and Pharmacodynamics of FOR-6219		Completed	no	Drug: Placebo|Drug: FOR-6219	Forendo Pharma Ltd.|Richmond Pharmacology Limited
Endometriosis	NCT04686669		A Relative Bioavailability Study of FOR-6219 in Capsule and Tablet Formulations		Completed	no	Drug: FOR-6219 capsule formulation|Drug: FOR-6219 tablet formulation	Forendo Pharma Ltd.|Richmond Pharmacology Limited

Clinical trial NCT00997373 evaluated Letrozole treatment (2.5 mg/day) as parallel assignment in 24 patients with grade 1 or grade 2 endometrial cancer 2–3 weeks before hysterectomy or repeat biopsy. This study explored changes at Ki-67 expression in tissue samples and found decreased expression of this proliferation marker after treatment with the aromatase inhibitor.

Two trials chaired by Dr. Slomovitz explored the efficacy of combining an aromatase inhibitor with an inhibitor of the mTOR signalling. Phase 2, single group assignment trial NCT01068249 (Slomovitz et al., 2015) evaluated the clinical benefit and the safety of the mTOR inhibitor Everolimus (10 mg/day) combined with Letrozole (2.5 mg/day) in 38 patients with recurrent endometrial cancer at 8 weeks of treatment, then every 12 weeks, up to 2 years. In total, 35 patients completed this trial with progression free survival of 3 months (95% confidence interval −95%CI-: 1.9–15.7) and overall survival of 14 months (95%CI: 9.5–24.4). Nine patients showed complete response and two patients had partial response. The second phase 2 trial NCT02228681 evaluated the effectiveness of the mTOR inhibitor Everolimus (10 mg/day) and Letrozole (2.5 mg/day) compared with Tamoxifen (20 mg/day) and Medroxyprogesterone acetate (200 mg/day) and severity of side effects in 74 women with advanced, recurrent or persistent endometrial cancer (FIGO stage III or IV). Longer disease-free survival was seen in patients treated with the first drug combination, with a median of 6.4 months and 95%CI of 3.8–17.7 versus 3.7 months (95%CI: 2.5-8.9). However, a higher frequency of adverse side effects, categorised as grade 3 using Common Toxicity Criteria for Adverse Events, was noticed in the Everolimus/Letrozole arm (73%) compared with the Tamoxifen/Medroxyprogesterone group (43.2%).

Another phase 2 trial, NCT02657928 (Colon-Otero et al., 2020), investigated the effects of combined four-week treatment with cyclin-dependent-kinase (CDK) 4 and CDK6 inhibitor Ribociclib (400 mg/day) and Letrozole (2.5 mg/day) in 20 patients with relapsed ER positive endometrial cancer. This study aimed to compare the results to previous clinical trials evaluating Letrozole treatment. After 12 weeks, 57.9% of the patients were alive and free of progression, progression free survival was 5.4 months (95%CI: 3.1–11.8) and overall survival was 15.7 months.

#### 2.2.3 Aromatase inhibitors and ovarian cancer

Aromatase inhibitors have been studied in ovarian cancer patients in 41 trials, seven completed, five terminated and for five trials, results are available (Table 2). Phase 2 trial NCT00505661 investigated the efficiency of Letrozole (2.5 mg/day) in 12 patients with advanced or recurrent borderline tumours or low-grade epithelial cancers from the ovary, fallopian tube or peritoneum. The primary outcome of this trial was Objective Response Rate Following Treatment With Letrozole. Only nine patients were included, where three had stable disease and five progressed in two-year time. This study was terminated due to slow patient recruitment.

Phase 2/3 trial NCT02101788 (Gershenson et al., 2022) compared treatment with mitogen-activated protein kinase (MEK) inhibitor Trametinib with standard treatments (either Letrozole, Tamoxifen, Paclitaxel, pegylated liposomal Doxorubicin, or the topoisomerase inhibitor Topotecan) in patients with recurrent, progressive or metastatic low-grade ovarian cancer. Patients treated with Trametinib had longer progression free survival (13 months, 95%CI: 9.9–15.0) and overall survival (37 months, 95%CI: 30.3 to NA) as compared with patients treated with standard care (progression free survival: 7.2 months, 95%CI: 85.6–9.9; overall survival: 29.2 months, 95%CI: 23.5–51.6).

The phase 2 trial NCT02657928 (Colon-Otero et al., 2020) investigated the effects of combined four-week treatment with the CDK4/CDK6 inhibitor Ribociclib (400 mg/day) and Letrozole (2.5 mg/day) in 20 patients with ER positive ovarian cancer. After 12 weeks, 52.6% of patients were alive and free of progression, progression free survival was 2.8 months (95%CI: 2.6–9.1) and overall survival was 18.9 months.

Phase 2, single group assignment trial NCT02283658 aimed at evaluating the efficacy of Letrozole combined with the mTOR inhibitor Everolimus. In total, 19 out of the 20 recruited patients completed the trial and 47% (95%CI: 24-71) were free of disease at 12 weeks (progression free survival: 3.9 months, 95%CI: 2.8 - 11), 21% (95%CI 6-46) had a therapeutic response based on CA-125 level (CA-125 response, defined as a 50% or greater reduction in baseline CA-125).

#### 2.2.4 Aromatase inhibitors and endometriosis

Aromatase inhibitors were investigated also in patients with endometriosis, nine trials have been registered, seven completed and for one trial, NCT02203331, results are known (Table 2). This randomized, double-blind, parallel-group, Phase 2b trial determined the efficacy and safety of different dose combinations of an aromatase inhibitor (Anastrozole) and progestin (Levonorgestrel) in an intravaginal ring versus placebo and Leuprorelin/Leuprolide acetate as a new treatment option for patients with endometriosis-associated pelvic pain. This trial included 319 patients and found no statistical difference in pain between different treatments. The use of aromatase inhibitors for the treatment of endometriosis is thoroughly reviewed in the clinical Endometriosis Guidelines issued by the European Society of Human Reproduction and Embryology (updated in 2022; (Becker et al., 2022).

#### 2.2.5 Aromatase inhibitors and prostate cancer

Six clinical studies investigated effects of aromatase inhibitors in prostate cancer patients and two were completed, but the results of these studies have not yet been published.

## 3 Sulfatase pathway in hormone dependent diseases

The gene encoding for *STS* is expressed in female and male reproductive organs (Mueller et al., 2015; Sinreih et al., 2017; Konings et al., 2018a). Higher *STS* levels were reported in primary breast cancer and also in soft tissue metastases (Hormozian et al., 2007; Poisson Pare et al., 2009). In patients with ER positive breast cancer, *STS* expression was associated with lymph node metastases, higher grade and poor prognosis (Miyoshi et al., 2003). However, a more recent study reported associations between higher *STS* expression and lower risk for relapse and distant metastases in a cohort of 139 breast cancer patients (McNamara et al., 2018).

In endometrial cancer, studies that compared cancer tissue with adjacent morphologically normal tissue reported unchanged (Smuc and Rizner, 2009; Rizner, 2013; Sinreih et al., 2017; Cornel et al., 2018; Cornel et al., 2019) or increased *STS* expression (Lepine et al., 2010). However, STS protein levels did not associate with progression free or overall survival in 59 patients with endometrial cancer (Lee et al., 2016). STS was also detected in different types of ovarian cancer, clear cell, serous and mucinous carcinomas using immunohistochemistry (Okuda et al., 2001), without significant difference seen in expression between serous ovarian cancer and ovarian surface epithelial tissue (Ren et al., 2015). In advanced stage ovarian cancers, high STS activity associated with poor progression free survival (Chura et al., 2009). Also SULT1E1 was detected in epithelial ovarian cancer (Calvillo-Robledo et al., 2021) with significantly higher immunohistochemical levels seen in low grade cancers *versus* high grade cancers. In these high grade serous ovarian cancers, SULT1E1 was associated with a better overall survival (Mungenast et al., 2017). In prostate cancer, STS and SULT1E1 were detected in 85% and 75% of the cases, respectively by immunohistochemistry (Nakamura et al., 2006). STS was found to be overexpressed in patients resistant to treatment with AR antagonists (Enzalutamide) or CYP17A1 inhibitors (Abiraterone) (Armstrong et al., 2020). STS is expressed also in endometriosis (ovarian, peritoneal and infiltrating) with high levels seen in ovarian endometriosis (Rizner, 2009), superficial and deep-infiltrating endometriosis (Piccinato et al., 2016; Piccinato et al., 2018; Da Costa et al., 2022).

Estrone-S has relatively high blood concentrations (0.11–1.84 nM) in postmenopausal women (Audet-Walsh et al., 2011) and middle-aged men (1.53–1.72 nM) (Giton et al., 2011) and can serve as a circulating reserve for intracellular activation to free estrone *via* the sulfatase pathway. This is supported by significantly higher estrone-S levels in breast tumour tissues compared with plasma (Pasqualini et al., 1997).

In tissues and cells that express estrone-S transporters from the OATP superfamily (Hagenbuch and Meier, 2003) and STS, estrone-S can enter cells and is metabolised to estrone. The intracellular levels of estrone depend on the balance between STS and SULT1E1, which catalyses sulfation of estradiol and estrone. Furthermore, formation of estradiol depends on expression of HSD17B1 and the ratio between the reductive HSD17B1 and the oxidative HSD17B2.

### 3.1 Overview of sulfatase inhibitors

In the last decades, a series of STS inhibitors has been synthetized and tested in cell based and animal models. The field of STS inhibitors has been covered by several reviews (Maltais and Poirier, 2011; Poirier, 2015; Thomas and Potter, 2015; Anbar et al., 2021) and will not be addressed in detail here. STS inhibitors include steroidal and non-steroidal compounds and can be divided into sulfomoylated and non-sulfomoylated compounds (Maltais and Poirier, 2011). The non-steroidal STS inhibitor STX64 also referred as Irosustat was the first to enter the clinical phase in postmenopausal women with breast cancer while steroidal inhibitor (E1-3-O-sulfamate, EMATE) together with progestin norethindrone acetate has been studied in endometriosis patients (Pohl et al., 2014). Clinical trials examining efficacy of STS inhibitors are described below.

### 3.2 Clinical trials targeting sulfatase

Based on information available at the database Clinical Trials, inhibitors of the sulfatase have been tested in patients with breast cancer, endometrial cancer, and patients with endometriosis.

#### 3.2.1 STS inhibitors and breast cancer

In breast cancer the first phase 1 clinical trial with an STS inhibitor has been performed in 2003 and 2004 (Stanway et al., 2006). Fourteen postmenopausal patients with locally advanced or metastatic disease who concluded at least one form of systemic treatment received either 5 or 20 mg of Irosustat as initial dose followed by weakly cycles, consisting of daily doses for 5 days followed by 9 days washout period. Inhibition of STS activity was determined in peripheral blood lymphocytes and tumour tissue with median values of 98% and 99% seen after five-day treatment, respectively. Treatment with STS inhibitor decreased serum levels of estrone, estradiol, androstenediol, DHEA but also androstenedione and testosterone (Stanway et al., 2006).

After this first phase 1 clinical trial, seven trials were registered in Database Clinical Trials and two have been completed (NCT01840488, NCT01785992; Table 2). The aims of the first study, were; i) to determine the optimal biological dose and recommended dose of Irosustat (BN83495) in postmenopausal women with ER positive locally advanced or metastatic breast cancer with disease progression after prior hormonal therapy; and ii) to provide information on safety and dose response, when the drug was given by repeated once daily oral administrations (Coombes et al., 2013). This trial was performed in three parts with a seven-day observation period after the first dose, 28-day period with daily doses and a continuation period as long as there was a benefit for the patient. The whole study included 50 patients. Five doses of Irosustat were tested and a dose of 40 mg was established as optimal biological and recommended dose (Coombes et al., 2013).

The second, phase 2 clinical trial, IRIS, evaluated the safety and effectiveness of Irosustat when added to an aromatase inhibitor in breast cancer patients. A study group of 27 postmenopausal women with ER positive locally advanced or metastatic breast cancer who progressed after first line treatment with an aromatase inhibitor received 40 mg of Irosustat once daily in addition to the aromatase inhibitor. The study showed clinical benefit with the median progression free survival of 2.7 months (2.5–4.6 95%CI) and acceptable safety profile (Palmieri et al., 2017).

Two phase 2 trials in breast cancer patients were prematurely terminated (NCT01230970, NCT01662726). The first trial started in 2011 and aimed to assess the short term intra-tumoral and peripheral effects of Irosustat administered for 14 days prior to surgery in postmenopausal women with newly diagnosed primary invasive ER positive breast cancer. This study terminated after recruiting only two patients due to the futility analysis. The second trial IPET investigated the effects of Irosustat on growth of breast cancer as a primary endpoint. Postmenopausal women with early, hormone sensitive, treatment naïve breast cancer received 40 mg of Irosustat once daily for 2 weeks. The effects of Irosustat were evaluated by PET scans (Positron Emission Tomography). Secondary endpoints were the assessment of pharmacodynamics profile and safety and tolerability of Irosustat. Thirteen patients were recruited but the study was unfortunately terminated prematurely due to challenging recruitment.

#### 3.2.2 STS inhibitors and endometrial cancer

Two clinical trials in endometrial cancer patients are registered in the database Clinical Trials (NCT00910091 and NCT01251354; Table 2). Randomised controlled trial NCT00910091 was conducted using the STS inhibitor Irosustat at a dose of 40 mg/day in comparison with Megestrol acetate (160 mg/day) in 73 patients with advanced or metastatic disease. The study population consisted of patients with mainly endometrioid endometrial cancers (61.1% and 64.9% in the two arms, respectively) grade I or II (69% and 75%) with documented ER positivity in primary or metastatic cancer without prior systemic treatment with exception of adjuvant chemotherapy. This phase 2 multicentre study has been stopped after futility analysis. The results are available and have been published (Pautier et al., 2017). Lower percentage of patients treated with Irosustat showed no progression and did not die 6 months after treatment (36.1%) compared with patients treated with Megestrol acetate (54.1%). The median progression free survival was 16.1 weeks versus 40.1 weeks and clinical benefit was achieved in 57.1% and 70.6% of patients treated with Irosustat and Megestrol acetate, respectively. There was no significant difference in the number of adverse effects between these treatment groups. The authors commented that the lower efficacy of Irosustat may have been associated with lower percentage of progesterone receptor positive tumours in this treatment group compared with Megestrol acetate group but have not questioned potential difference in the levels of sulfatase which is targeted by Irosustat. The authors concluded that Irosustat is not efficacious as a single therapy and should be investigated further in combination with other hormonal agents (Pautier et al., 2017). As a consequence of the futility analysis of this study, the second single group assignment, open label trial NCT01251354 was terminated after enrolling six patients.

#### 3.2.3 STS inhibitors and endometriosis

One phase 2 clinical trial explored the use of an STS inhibitor in endometriosis patients (NCT01631981; Table 2). STS inhibitor PGL 2001 (estradiol sulfamate, E2MATE) was tested in 162 patients with endometriosis with concomitant administration of progestin NETA (Norethisterone Acetate). The effects of treatment of symptoms related with endometriosis were evaluated and the study was concluded 2013, but the results have not yet been published. It should be noted that E2MATE has estrogenic effects in rodents, and such effects could be elicited in humans as well. Therefore, Irosustat could have been a better choice than E2MATE for testing in patients with endometriosis, a disease characterised by estrogen-dependent growth of endometrium like tissue outside uterine cavity.

#### 3.2.4 STS inhibitors and prostate cancer

One phase 1, singe group assignment trial is registered at the database Clinical Trials (Table 2). and assessed the pharmacodynamics and safety of escalating doses of Irosustat in 17 prostate cancer patients non-responsive to antiandrogen drugs. The study is completed, but no results are available.

## 4 HSD17B1 and the HSD17B1:HSB17B2 redox balance in hormone dependent diseases

As explained earlier, the enzymes HSD17B1 and HSD17B2 control the last step in the local formation of estradiol. Both enzymes are expressed in a variety of tissues, including female and male reproductive tissues, bone, lungs, gastrointestinal tract, central nervous system (Konings et al., 2018b; Heinosalo et al., 2019). HSD17B1 regulation and its enzymatic activity towards substrates other than estrone have been recently reviewed (Heinosalo et al., 2019). Deviated expression of *HSD17B1* and *HSD17B2* are reported in various estrogen dependent conditions. Results in the context of endometrial pathologies in terms of up or downregulation compared to healthy tissues are inconsistent (Sinreih et al., 2017; Konings et al., 2018c; Cornel et al., 2019), however, high levels of *HSD17B1* and low levels of *HSD17B2* are associated with poor prognosis in endometrial carcinoma (Cornel et al., 2012). In addition, high expression of *HSD17B1* is associated with deviated follicular fluid steroid levels in polycystic ovarian syndrome (Yu et al., 2021), foetal growth restriction (Zhu et al., 2018), chronic obstructive pulmonary disease (COPD; (Konings et al., 2017); and other disorders including lung cancer, skin and eye diseases (Konings et al., 2018a; Heinosalo et al., 2019). Additionally, genetic variants in the *HSD17B1* gene are associated with amyotrophic lateral sclerosis (Boddy et al., 2022), endometriosis associated infertility (Egashira et al., 2022) and other disorders (Konings et al., 2018b). Animal studies as well point out that expression of *HSD17B1* leads to endometrial hyperplasia (Saloniemi et al., 2010), endometrial cancer (Konings et al., 2018b; Xanthoulea et al., 2021), adenomyosis (Heinosalo et al., 2022), and mammary gland pathologies (Jarvensivu et al., 2018).

### 4.1 Overview of HSD17B1 inhibitors

In the last decades, several compounds were developed and explored for their ability to block the HSD17B1 enzyme (Brozic et al., 2008; Niinivehmas et al., 2018; Abdelsamie et al., 2019; Herman et al., 2019; Kulmany et al., 2021; Mottinelli et al., 2021), and several molecules were patented (Poirier, 2010; 2015).

Targeting HSD17B1 is particularly challenging because a good inhibitor must have no estrogenic effect. In addition, they must be specific towards HSD17B1 and have no inhibitory action towards other HSD17B enzymes. Finally, HSD17B1 orthologs show important differences in substrate specificity, tissue distribution and amino-acid sequences, which makes the preclinical *in-vivo* studies very complex since a compound with good inhibitory action against the human HSD17B1 may have no activity on the rodent enzyme (Moller et al., 2010). As a consequence, most *in-vivo* studies performed so far (see below) were executed with xenografts or transgenic animals expressing the human enzyme (Saloniemi et al., 2010; Konings et al., 2018b; Konings et al., 2018c; Jarvensivu et al., 2018; Xanthoulea et al., 2021; Heinosalo et al., 2022).

As for other enzymes, HSD17B1 inhibitors can be steroidal (derivatives of estrone or estradiol) and non-steroidal, where molecules mimic the steroid scaffold (Konings et al., 2018b). In case of steroidal HSD17B1 inhibitors, carbons 6, 15, 16, and 17 of the steroid backbone have been modified. The team of Dr. Poirier developed a series of estradiol derivatives with C16 beta-m-carbamoylbenzyl modifications (Lesperance et al., 2021). CC-156, the first developed, showed some estrogenic action, which was eliminated upon C3-bromoethyl modification, leading to compound PBRM and subsequent analogues. The inhibitory potency of the lead molecule has been improved displaying EC50 as low as 68 nM *in-vitro* on monolayer cells (Lesperance et al., 2021). Further, the authors were able to add STS inhibitory properties to the molecule by including one or two sulfamate groups in the steroid D-ring (Lesperance et al., 2021). The therapeutic activity of PBRM was recently tested *in-vivo* in a mouse model of breast cancer (Poirier et al., 2021). Tumour xenografts were induced subcutaneously using the T47D breast cancer cell line. Mice were ovariectomised, treated with estrone with or without PBRM and drug treatment significantly decreased the tumour size. Furthermore, since PBRM is an irreversible inhibitor of HSD17B1, the therapeutic effects were also evident after reducing the frequency of drug administration (Lesperance et al., 2021; Poirier et al., 2021). More recently, the same team showed good efficacy of this compound in endometriosis using ex-patients/*ex-vivo* experiments, although they failed to show therapeutic responses in a primate model of endometriosis, also due to the fact that their experiment was underpowered (Poirier et al., 2022).

A series of compounds based on the estrone backbone with C15 modification was developed during the first decade of 2000 (Messinger et al., 2009). Based on the 3D crystal structure studies, once the substrate is docked in the catalytic site, C15 is exposed outside the enzyme-substrate complex and is therefore amenable for modification to improve chemical and pharmacological properties. In addition, C15 is engaged in ER-ligand complex, suggesting that modification in this carbon would prevent any unwanted estrogenic action (Messinger et al., 2009). The therapeutic efficacy of this compound was thoroughly tested in *in-vivo* models of various conditions. Husen et al. induced breast cancer by subcutaneous injection of MCF7 cells in mice. Animals were ovariectomised and estrone with or without the inhibitor was delivered using osmotic minipumps. The authors observed a reduction in breast cancer growth of over 80% (Husen et al., 2006a). The same compound or its derivatives showed efficacy in orthotopic models of breast (Jarvensivu et al., 2018), endometrial cancer (Xanthoulea et al., 2021), in a mouse model of endometrial hyperplasia (Saloniemi et al., 2010) and in various models of endometriosis, based on *ex-vivo* patient biopsies (Delvoux et al., 2014) or based on primate models (Einspanier et al., 2006; Arnold and Einspanier, 2013). In these last studies, marmoset monkeys with endometriosis were treated daily with the drug or with placebo for 4 weeks and were compared with six control monkeys without disease. The authors could show that endometriosis impaired the social behaviour (social grooming) and increased stress (decreased time in a hammock) in these animals, and that treatment with the HSD17B1 inhibitor restored these conditions to the levels seen in controls animals. No additional cognitive effects were seen upon drug treatment (Arnold and Einspanier, 2013). In a subsequent study, the same drug regiment showed to reduce the number of lesions in treated compared with untreated animals. Interestingly, drug treatment did not affect the ovarian cycle (Einspanier et al., 2006). Organon Finland further improved the pharmacology of this compound which is currently in the clinical study phase for endometriosis (see next paragraph).

An alternative approach to inhibit HSD17B1 that overcomes the problem of estrogenic activity or any other off target effect is the use of siRNA mediated downregulation of the HSD17B1 enzyme. Such elegant approach was recently tested in a mouse model of breast cancer (Li et al., 2019).

### 4.2 Clinical trials targeting HSD17B1

Only the C15 estrone derivative developed by Organon Finland, former Forendo pharma (compound FOR-6219/OR-6219) reached the clinical phase for endometriosis with three clinical trials registered in the database Clinical Trails (Table 2). Phase 1 and 1b trials NCT04686669 and NCT03709420 determined the bio-availability of the compound administered orally as gelatine capsule in 12 subjects (NCT04686669) and then the safety, tolerability, food interactions, the pharmacokinetics and pharmacodynamics of escalating doses of the drug in 87 subjects (NCT03709420). The phase 2 randomized, double-blind, Elena study (NCT05560646) is currently recruiting patients and aims at evaluating the efficacy and safety of OG-6219 in women with moderate to severe endometriosis (https://www.elenaendometriosisstudy.com/#!/).

## 5 Perspectives

Targeting the local generation of steroid hormones represents a novel and promising approach for hormone dependent conditions as it is now clear that a large part of the hormones that sustain disease conditions are generated locally from inactive precursors and via the use of alternative routes that are not targeted by current medications. For instance, DHEA-S is a depot for local androgen synthesis in prostate cancer patients also after hormone therapy using 17a-hydroxylase-17,20-lyase (CYP17A1) inhibitors (Penning, 2018), and STS inhibitors demonstrated useful for the treatment of prostate cancer resistant to anti-androgen drugs (Armstrong et al., 2020). Similar mechanisms may be present in estrogen dependent conditions using estrone-S and estradiol-S as reservoir.

The development of resistance to hormone-deprivation therapy is a major concern, and combination of drugs instead of monotherapy can extend the sensitivity period. Studies have also shown that inhibition of aromatase can increase the expression and activity of STS in breast cancer (Foster, 2021). Therefore, the development of dual inhibitors targeting multiples steps in the intracrine network is a useful and promising approach. Effective molecules have been developed, like the dual aromatase-sulfatase inhibitor STX681 (Foster et al., 2008), and other inhibitors (Woo et al., 2010; Woo et al., 2011; Harrelson and Lee, 2016) and the sulfatase-HSD17B1 inhibitor (Mohamed et al., 2022). Also the approach of combining pre-receptor (or intracrine) drugs and receptor-blocking agents deserves attention in the future to obtain a complete shut down of the hormone signalling. Interestingly, molecules with dual pre-receptor (5-alpha-reducates) and receptor inhibition (androgen receptor) were recently developed (Lao et al., 2019).

Hormonal drugs require effective hormone signalling, and other intracellular signalling pathways (such as Akt-PI3K-mTOR) can compromise the response to hormonal drugs by taking over the proliferative actions (van Weelden et al., 2019). In this context, several clinical trials are currently underway to investigate the combination of hormonal with other targeted treatments and the added values of these approaches. Overall, although novel drugs and new knowledge on their therapeutic efficacy is continuously generated, it is unfortunate that the results of a number of concluded trials are neither published nor disclosed.

An important aspect of the use of hormonal drugs is that not all patients may be responsive, especially (but not only) in the field of oncology. In this context, it is becoming increasingly clear how to predict a response to hormonal drugs in patients, which will help to target the treatments to responsive patients only (Verhaegh et al., 2014; Inda et al., 2020; van Weelden et al., 2021).

For the future, full characterisation of disease features by immunohistochemistry, and molecular analyses or biomarkers is needed to stratify patients based on expression of the drug target, likelihood of response to treatment and risk of recurrence and medical treatment.

Drugs targeting hormone action have been introduced decades ago, but identification of new drug targets, dual action drugs, combination with other targeted treatments can provide benefit to the cancer patients and patients with other non-oncological but still distressing conditions.

## Abbreviations

BED, bromodomain and extra-terminal motif; CDK, cyclin dependent kinase; COX-2, cyclooxygenase 2; FGFR, fibroblast growth factor receptor; HDAC, histone deacetylase; HER2, human epidermal growth factor receptor 2; IGFI, insulin like growth factor; mTOR, mammalian target of rapamycin; PD-1, programmed cell death protein 1; PI3K, phosphoinositide 3-kinases; TK, tyrosine kinase; VEGF, vascular endothelial growth factor.
