# Supplementary material for: Targeting the formation of estrogens for treatment of hormone dependent diseases–current status
Source: Front Pharmacol. 2023 Apr 28;14:1155558. doi: 10.3389/fphar.2023.1155558 (PMC10175629; doi:10.3389/fphar.2023.1155558)
Supplement: Supplementary file 1 [file Table1.DOCX]

**Supplemental Table I:** Overview of the clinical studies on breast cancer registered in the database Clinical Trials and using an aromatase inhibitor in combination with any other targeted treatment.

| **NCT Number**  **Reference** | **Title** | **Acronym** | **Target *** | **Phase** | **Subjects** | **start-completion** |
| --- | --- | --- | --- | --- | --- | --- |
| NCT01720602 | Vorinostat in Treating Patients With Stage IV Breast Cancer Receiving Hormone Therapy |  | HDAC inhibitor | Not Applicable | 15 | 2012-2020 |
| NCT01153672 | Vorinostat in Treating Patients With Stage IV Breast Cancer Receiving Aromatase Inhibitor Therapy |  | HDAC inhibitor | Not Applicable | 8 | 2010-2016 |
| NCT02392611 | Study to Evaluate Safety, Tolerability, Pharmacokinetics, and Pharmacodynamics of Alobresib (Formerly GS-5829) in Adults With Advanced Solid Tumors and Lymphomas and in Combination With Exemestane or Fulvestrant in Adults With Estrogen Receptor Positive Breast Cancer |  | BET inhibitor | Phase 1 | 33 | 2015-2017 |
| NCT00217399 | Sorafenib and Anastrozole in Treating Postmenopausal Women With Metastatic Breast Cancer |  | CDK4/6 inhibitor | Phase 1/2 | 35 | 2005-2013 |
| NCT02732119 | Study of Ribociclib With Everolimus + Exemestane in HR+ HER2- Locally Advanced/Metastatic Breast Cancer Post Progression on CDK 4/6 Inhibitor. | TRINITI-1 | CDK4/6 inhibitor | Phase 1/2 | 104 | 2016-2020 |
| NCT02871791 | Palbociclib With Everolimus + Exemestane In BC |  | CDK4/6 inhibitor | Phase 1/2 | 41 | 2016-2021 |
| NCT01202591  (Coombes et al., 2022) | Safety and Efficacy of AZD4547 in Combination With Fulvestrant vs. Fulvestrant Alone in ER+ Breast Cancer Patients | GLOW | FGFR1,2,3 inhibitor | Phase 1/2 | 127 | 2010-2014 |
| NCT01791985 | AZD4547 & Anastrozole or Letrozole (NSAIs) in ER+ Breast Cancer Patients Who Have Progressed on NSAIs (RADICAL) | RADICAL | FGFR1,2,3 inhibitor | Phase 1/2 | 52 | 2012-2018 |
| NCT01105312 | Panobinostat and Letrozole in Treating Patients With Metastatic Breast Cancer |  | HDAC inhibitor | Phase 1/2 | 28 | 2010-2013 |
| NCT01216176 | A Pharmacokinetic and Randomized Trial of Neoadjuvant Treatment With Anastrozole Plus AZD0530 in Postmenopausal Patients With Hormone Receptor Positive Breast Cancer |  | TK inhibitor | Phase 1/2 | 71 | 2008-2018 |
| NCT02366130 | Trial of Ra-223 Dichloride in Combination With Hormonal Therapy and Denosumab in the Treatment of Patients With Hormone-Positive Bone-Dominant Metastatic Breast Cancer |  | radiopharmaceutical | Phase 2 | 45 | 2015-2020 |
| NCT00721409 | Study Of Letrozole With Or Without Palbociclib (PD-0332991) For The First-Line Treatment Of Hormone-Receptor Positive Advanced Breast Cancer |  | CDK4/6 inhibitor | Phase 2 | 177 | 2008-2017 |
| NCT01684215 | A Study Of Oral Palbociclib (PD-0332991), A CDK4/6 Inhibitor, As Single Agent In Japanese Patients With Advanced Solid Tumors Or In Combination With Letrozole For The First-Line Treatment Of Postmenopausal Japanese Patients With ER (+) HER2 (-) Advanced Breast Cancer |  | CDK4/6 inhibitor | Phase 2 | 61 | 2012-2018 |
| NCT02040857 | Palbociclib in Combination With Adjuvant Endocrine Therapy for Hormone Receptor Positive, HER2 Negative Invasive Breast Cancer |  | CDK4/6 inhibitor | Phase 2 | 162 | 2014-2020 |
| NCT02630693 | Two Different Schedules of Palbociclib + Second Line Endocrine Therapy in Estrogen Receptor Positive, HER2 Neg Advanced/Metastatic Breast Cancer |  | CDK4/6 inhibitor | Phase 2 | 180 | 2015-2019 |
| NCT02441946 | A Neoadjuvant Study of Abemaciclib (LY2835219) in Postmenopausal Women With Hormone Receptor Positive, HER2 Negative Breast Cancer | Neo MONARCH | CDK4/6 inhibitor (Neoadjuvant) | Phase 2 | 224 | 2015-2018 |
| NCT02296801 | A Phase II Randomized Study Evaluating the Biological and Clinical Effects of the Combination of Palbociclib With Letrozole as Neoadjuvant Therapy in Post-Menopausal Women With Estrogen-Receptor Positive Primary Breast Cancer | PALLET | CDK4/6 inhibitor (Neoadjuvant) | Phase 2 | 307 | 2015-2019 |
| NCT00038103 | Open-Label Study Of Exemestane With Or Without Celecoxib In Postmenopausal Women With ABC Having Progressed On Tamoxifen |  | COX-2 inhibitor | Phase 2 | 111 | 2002-2008 |
| NCT00201773 | Exemestane With Celecoxib as Neoadjuvant Treatment in Postmenopausal Women With Stage II, III, and IV Breast Cancer |  | COX-2 inhibitor | Phase 2 | 22 | 2003-2011 |
| NCT00708214 | BIBW 2992 and Letrozole in Hormonoresistant Metastatic Breast Cancer |  | dual EGFR/HER2 inhibitor | Phase 2 | 28 | 2007- |
| NCT00422903 | Letrozole In Combination With Lapatinib In Neoadjuvant Treatment Of Early Breast Cancer |  | dual EGFR/HER2 inhibitor | Phase 2 | 92 | 2007-2011 |
| NCT00828854 | Study of the Effect of the Addition of SNDX-275 (Entinostat) to Continued Aromatase Inhibitor (AI) Therapy in Postmenopausal Women With ER+ Breast Cancer Whose Disease is Progressing |  | HDAC inhibitor | Phase 2 | 27 | 2008-2009 |
| NCT00676663 | Study to Evaluate Exemestane With and Without Entinostat (SNDX-275) in Treatment of Postmenopausal Women With Advanced Breast Cancer | ENCORE301 | HDAC inhibitor | Phase 2 | 130 | 2008-2012 |
| NCT00482391 | Doxorubicin and Cyclophosphamide Followed by Paclitaxel, Trastuzumab, and Lapatinib in Treating Patients With HER2/Neu-Overexpressed Breast Cancer |  | HER2 + TK inhibitor | Phase 2 | 95 | 2007-2011 |
| NCT01491737 | A Study of Pertuzumab in Combination With Trastuzumab Plus an Aromatase Inhibitor in Participants With Metastatic Human Epidermal Growth Factor Receptor 2 (HER2)-Positive and Hormone Receptor-Positive Advanced Breast Cancer | PERTAIN | HER2 inhibitor | Phase 2 | 258 | 2012-2019 |
| NCT01151046 | Trial of Exemestane +/- MM-121 in Postmenopausal Women With Locally Advanced or Metastatic Estrogen Receptor Positive and/or Progesterone Receptor Positive Her2 Negative Breast Cancer |  | HER3 inhibitor | Phase 2 | 118 | 2010-2014 |
| NCT03659136 | The XENERA‚Ñ¢ 1 Study Tests Xentuzumab in Combination With Everolimus and Exemestane in Women With Hormone Receptor Positive and HER2-negative Breast Cancer That Has Spread |  | IGF-1/IGF-2 + mTOR inhibitors | Phase 2 | 103 | 2018-2022 |
| NCT00728949 | A Study for Safety and Effectiveness of IMC-A12 by Itself or Combined With Antiestrogens to Treat Breast Cancer |  | IGF-IR inhibitor | Phase 2 | 93 | 2008-2015 |
| NCT01446159 | Study of MEDI-573 Plus Standard Endocrine Therapy for Women With Hormone-sensitive Metastatic Breast Cancer |  | IGFI/IGFII inhibitor | Phase 2 | 188 | 2011-2019 |
| NCT01605396 | A Phase II Trial of Ridaforolimus and Exemestane, Compared to Ridaforolimus, Dalotuzumab and Exemestane in Participants With Breast Cancer (MK-8669-064) |  | mTOR + IGF1R inhibitors | Phase 2 | 80 | 2012-2018 |
| NCT00062751 | Study Evaluating Temsirolimus (CCI-779) In Breast Neoplasms |  | mTOR inhibitor | Phase 2 | 108 | 2002-2009 |
| NCT00570921 | Study of Combined Fulvestrant and Everolimus in Advanced/Metastatic Breast Cancer After Aromatase Inhibitor Failure | BRE-43 | mTOR inhibitor | Phase 2 | 33 | 2008-2015 |
| NCT01231659 | Safety and Efficacy of RAD001 (Everolimus) in Combination With Letrozole in the Treatment of Postmenopausal Women With Locally Advanced or Metastatic Breast Cancer |  | mTOR inhibitor | Phase 2 | 72 | 2011-2015 |
| NCT01783444 | A Phase II Study of Everolimus in Combination With Exemestane Versus Everolimus Alone Versus Capecitabine in Advance Breast Cancer. | BOLERO-6 | mTOR inhibitor | Phase 2 | 309 | 2013-2018 |
| NCT01698918 | Open-label, Phase II, Study of Everolimus Plus Letrozole in Postmenopausal Women With ER+, HER2- Metastatic or Locally Advanced Breast Cancer | BOLERO-4 | mTOR inhibitor | Phase 2 | 202 | 2013-2021 |
| NCT02049957 | Safety and Efficacy Study of Sapanisertib in Combination With Exemestane or Fulvestrant in Postmenopausal Women With Estrogen Receptor Positive/Human Epidermal Growth Factor Receptor 2 Negative (ER+/HER2-) Metastatic Breast Cancer |  | mTOR inhibitor | Phase 2 | 118 | 2014-2018 |
| NCT02291913 | Everolimus Combined With Anti-estrogen Therapy in Hormone-Receptor-Positive HER-2 Negative Advanced Breast Cancer |  | mTOR inhibitor | Phase 2 | 48 | 2014-2019 |
| NCT02258451 | Study of Radium-223 Dichloride in Combination With Exemestane and Everolimus Versus Placebo in Combination With Exemestane and Everolimus in Subjects With Bone Predominant HER2 Negative Hormone Receptor Positive Metastatic Breast Cancer |  | mTOR inhibitor + radio-pharmaceutical | Phase 2 | 283 | 2015-2022 |
| NCT04075604 | A Study of Neoadjuvant Nivolumab + Palbociclib + Anastrozole in Post-Menopausal Women and Men With Primary Breast Cancer | CheckMate 7A8 | PD-1 + CDK4/6 inhibitors | Phase 2 | 23 | 2019-2021 |
| NCT02273973  (Saura et al., 2019) | A Study of Neoadjuvant Letrozole + Taselisib Versus Letrozole + Placebo in Post-Menopausal Women With Breast Cancer (LORELEI) |  | PI3K inhibitor (Neoadjuvant) | Phase 2 | 334 | 2014-2017 |
| NCT00338728 | Letrozole and Imatinib Mesylate in Treating Postmenopausal Participants With Estrogen or Progesterone Positive Metastatic Breast Cancer |  | TK inhibitor | Phase 2 | 59 | 2003-2018 |
| NCT00696072 | Randomized Phase II Trial of Letrozole With or Without Dasatinib as First and Second-line Treatment for Hormone Receptor-positive, HER2-negative Post-menopausal Breast Cancer That is Unresectable, Locally Recurrent or Metastatic |  | TK inhibitor | Phase 2 | 120 | 2008-2014 |
| NCT00767520 | Safety and Efficacy of Exemestane Plus Dasatinib Versus Placebo for Advanced ER+ Breast Cancer |  | TK inhibitor | Phase 2 | 155 | 2009-2012 |
| NCT01466972 | Reversing Hormone Resistance in Advanced Breast Cancer With Pazopanib |  | TK inhibitor | Phase 2 | 30 | 2012-2029 |
| NCT00451555 | Enzastaurin Plus Fulvestrant vs. Placebo Plus Fulvestrant in Breast Cancer |  | VEGF inhibitor | Phase 2 | 156 | 2007-2018 |
| NCT00240071 | Study of Avastin (Bevacizumab) to Reverse Acquired Estrogen Independence in Previously Hormone Responsive Metastatic Breast Ca. |  | VEGF-A inhibitor | Phase 2 | 30 | 2005-2011 |
| NCT00405938 | Bevacizumab Given With Either Anastrozole or Fulvestrant With Trastuzumab for Postmenopausal Metastatic Breast Cancer |  | VEGF-A inhibitor | Phase 2 | 79 | 2006-2011 |
| NCT02692755  (Lynce et al., 2018) | Palbociclib / Letrozole or Fulvestrant in African American Women With HR+ HER2- Breast Cancer | PALINA | CDK4/6 inhibitor | Phase 2/3 | 35 | 2016-2021 |
| NCT02600923 | Palbociclib Plus Letrozole For Postmenopausal Women With HR(+) HER2(-) Advanced Breast Cancer For Whom Letrozole Is Deemed Appropriate |  | CDK4/6 inhibitor | Phase 3 | 131 | 2016-2019 |
| NCT03096847 | Study for Women and Men With Hormone-receptor Positive Locally Advanced or Metastatic Breast Cancer |  | CDK4/6 inhibitor | Phase 3 | 502 | 2016-2020 |
| NCT00022672 | A Study to Evaluate the Efficacy and Safety of Herceptin¬Æ (Trastuzumab) in Combination With Arimidex¬Æ (Anastrozole) an Aromatase Inhibitor Compared to Arimidex¬Æ Alone in Patients With Metastatic Breast Cancer |  | HER2 inhibitor | Phase 3 | 208 | 2001-2009 |
| NCT00073528 | Study Comparing Lapatinib (GW572016) And Letrozole Versus Letrozole In Subjects With Advanced Or Metastatic Breast Cancer |  | HER2 inhibitor | Phase 3 | 1286 | 2003-2018 |
| NCT00863655  (Baselga et al., 2012; Noguchi et al., 2014) | Everolimus in Combination With Exemestane in the Treatment of Postmenopausal Women With Estrogen Receptor Positive Locally Advanced or Metastatic Breast Cancer Who Are Refractory to Letrozole or Anastrozole | BOLERO-2 | HER2 inhibitor | Phase 3 | 724 | 2009-2014 |
| NCT01419197  (Kim et al., 2016; Krop et al., 2014; Krop et al., 2017) | A Study of Trastuzumab Emtansine in Comparison With Treatment of Physician's Choice in Participants With HER2-positive Breast Cancer Who Have Received at Least Two Prior Regimens of HER2-directed Therapy | TH3RESA | HER2 inhibitor | Phase 3 | 602 | 2011-2015 |
| NCT01160211  (Johnston et al., 2018) | A Study to Compare the Safety and Efficacy of an Aromatase Inhibitor in Combination With Lapatinib, Trastuzumab or Both for the Treatment of Hormone Receptor Positive, HER2+ Metastatic Breast Cancer | ALTERNATIVE | HER2 inhibitor | Phase 3 | 369 | 2011-2022 |
| NCT03176238 | Study in Post-menopausal Women With Hormone Receptor Positive, HER2-negative Advanced Breast Cancer | EVEREXES | HER2 inhibitor | Phase 3 | 235 | 2013-2019 |
| NCT00545077  (Martin et al., 2019) | Bevacizumab + Endocrine Treatment vs Endocrine Treatment as First Line in Postmenopausal Women |  | VEGF-A inhibitor | Phase 3 | 380 | 2007-2014 |
| NCT01743560 | An Open Label Study of Postmenopausal Women With Oestrogen Receptor Positive Locally Advanced or Metastatic Breast Cancer Treated With Everolimus (RAD001) With Exemestane, With Exploratory Epigenetic Marker Analysis | 4EVERUK | mTOR inhibitor | Phase 4 | 52 | 2013-2016 |
| NCT02679755  (Lynce et al., 2021) | Palbociclib In Combination With Letrozole As Treatment Of Post-Menopausal Women With HR+, HER2- Advanced Breast Cancer |  | CDK4/6 inhibitor | Phase 4 | 252 | 2016-2019 |

*** Abbreviations**

BED: bromodomain and extra-terminal motif

CDK: cyclin dependent kinase

COX-2: cyclooxygenase 2

FGFR: fibroblast growth factor receptor

HDAC: histone deacetylase

HER2: human epidermal growth factor receptor 2

IGFI: insulin like growth factor

mTOR: mammalian target of rapamycin

VEGF: vascular endothelial growth factor

PD-1: programmed cell death protein 1

PI3K: phosphoinositide 3-kinases

TK: tyrosine kinase

**Bibliography**

Baselga, J., Campone, M., Piccart, M., Burris, H.A., 3rd, Rugo, H.S., Sahmoud, T., Noguchi, S., Gnant, M., Pritchard, K.I., Lebrun, F., Beck, J.T., Ito, Y., Yardley, D., Deleu, I., Perez, A., Bachelot, T., Vittori, L., Xu, Z., Mukhopadhyay, P., Lebwohl, D., Hortobagyi, G.N., 2012. Everolimus in postmenopausal hormone-receptor-positive advanced breast cancer. N Engl J Med 366, 520-529.

Coombes, R.C., Badman, P.D., Lozano-Kuehne, J.P., Liu, X., Macpherson, I.R., Zubairi, I., Baird, R.D., Rosenfeld, N., Garcia-Corbacho, J., Cresti, N., Plummer, R., Armstrong, A., Allerton, R., Landers, D., Nicholas, H., McLellan, L., Lim, A., Mouliere, F., Pardo, O.E., Ferguson, V., Seckl, M.J., 2022. Results of the phase IIa RADICAL trial of the FGFR inhibitor AZD4547 in endocrine resistant breast cancer. Nat Commun 13, 3246.

Johnston, S.R.D., Hegg, R., Im, S.A., Park, I.H., Burdaeva, O., Kurteva, G., Press, M.F., Tjulandin, S., Iwata, H., Simon, S.D., Kenny, S., Sarp, S., Izquierdo, M.A., Williams, L.S., Gradishar, W.J., 2018. Phase III, Randomized Study of Dual Human Epidermal Growth Factor Receptor 2 (HER2) Blockade With Lapatinib Plus Trastuzumab in Combination With an Aromatase Inhibitor in Postmenopausal Women With HER2-Positive, Hormone Receptor-Positive Metastatic Breast Cancer: ALTERNATIVE. J Clin Oncol 36, 741-748.

Kim, S.B., Wildiers, H., Krop, I.E., Smitt, M., Yu, R., Lysbet de Haas, S., Gonzalez-Martin, A., 2016. Relationship between tumor biomarkers and efficacy in TH3RESA, a phase III study of trastuzumab emtansine (T-DM1) vs. treatment of physician's choice in previously treated HER2-positive advanced breast cancer. Int J Cancer 139, 2336-2342.

Krop, I.E., Kim, S.B., Gonzalez-Martin, A., LoRusso, P.M., Ferrero, J.M., Smitt, M., Yu, R., Leung, A.C., Wildiers, H., collaborators, T.R.s., 2014. Trastuzumab emtansine versus treatment of physician's choice for pretreated HER2-positive advanced breast cancer (TH3RESA): a randomised, open-label, phase 3 trial. Lancet Oncol 15, 689-699.

Krop, I.E., Kim, S.B., Martin, A.G., LoRusso, P.M., Ferrero, J.M., Badovinac-Crnjevic, T., Hoersch, S., Smitt, M., Wildiers, H., 2017. Trastuzumab emtansine versus treatment of physician's choice in patients with previously treated HER2-positive metastatic breast cancer (TH3RESA): final overall survival results from a randomised open-label phase 3 trial. Lancet Oncol 18, 743-754.

Lynce, F., Blackburn, M.J., Zhuo, R., Gallagher, C., Hahn, O.M., Abu-Khalaf, M., Mohebtash, M., Wu, T., Pohlmann, P.R., Dilawari, A., Tiwari, S.R., Chitalia, A., Warren, R., Tan, M., Shajahan-Haq, A.N., Isaacs, C., 2021. Hematologic safety of palbociclib in combination with endocrine therapy in patients with benign ethnic neutropenia and advanced breast cancer. Cancer 127, 3622-3630.

Lynce, F., Saleh, M., Shajahan-Haq, A., Gallagher, C., Dilawari, A., Hahn, O., Abu-Khalaf, M., Cai, L., Pohlmann, P., Mohebtash, M., Kamugisha, L., Isaacs, C., 2018. PALINA: A phase II safety study of palbociclib in combination with letrozole or fulvestrant in African American women with hormone receptor positive HER2 negative advanced breast cancer. Contemp Clin Trials Commun 10, 190-192.

Martin, M., Loibl, S., Hyslop, T., De la Haba-Rodriguez, J., Aktas, B., Cirrincione, C.T., Mehta, K., Barry, W.T., Morales, S., Carey, L.A., Garcia-Saenz, J.A., Partridge, A., Martinez-Janez, N., Hahn, O., Winer, E., Guerrero-Zotano, A., Hudis, C., Casas, M., Rodriguez-Martin, C., Furlanetto, J., Carrasco, E., Dickler, M.N., Group, G.S.B.C., Gbg, Alliance for Clinical Trials in, O., 2019. Evaluating the addition of bevacizumab to endocrine therapy as first-line treatment for hormone receptor-positive metastatic breast cancer: a pooled analysis from the LEA (GEICAM/2006-11_GBG51) and CALGB 40503 (Alliance) trials. Eur J Cancer 117, 91-98.

Noguchi, S., Masuda, N., Iwata, H., Mukai, H., Horiguchi, J., Puttawibul, P., Srimuninnimit, V., Tokuda, Y., Kuroi, K., Iwase, H., Inaji, H., Ohsumi, S., Noh, W.C., Nakayama, T., Ohno, S., Rai, Y., Park, B.W., Panneerselvam, A., El-Hashimy, M., Taran, T., Sahmoud, T., Ito, Y., 2014. Efficacy of everolimus with exemestane versus exemestane alone in Asian patients with HER2-negative, hormone-receptor-positive breast cancer in BOLERO-2. Breast Cancer 21, 703-714.

Saura, C., Hlauschek, D., Oliveira, M., Zardavas, D., Jallitsch-Halper, A., de la Pena, L., Nuciforo, P., Ballestrero, A., Dubsky, P., Lombard, J.M., Vuylsteke, P., Castaneda, C.A., Colleoni, M., Santos Borges, G., Ciruelos, E., Fornier, M., Boer, K., Bardia, A., Wilson, T.R., Stout, T.J., Hsu, J.Y., Shi, Y., Piccart, M., Gnant, M., Baselga, J., de Azambuja, E., 2019. Neoadjuvant letrozole plus taselisib versus letrozole plus placebo in postmenopausal women with oestrogen receptor-positive, HER2-negative, early-stage breast cancer (LORELEI): a multicentre, randomised, double-blind, placebo-controlled, phase 2 trial. Lancet Oncol 20, 1226-1238.
